# 1399. Dramatic Increase in Invasive Group A Streptococcal Infection in Persons Who Inject Drugs in an Urban Philadelphia Hospital

**DOI:** 10.1093/ofid/ofac492.1228

**Published:** 2022-12-15

**Authors:** Eric Altneu, Jessica Metlay, Hugh A Davis, Stephanie Spivack, Kaede Ota Sullivan, Sara K Schultz

**Affiliations:** Temple University Health System, Philadelphia, Pennsylvania; Lewis Katz School of Medicine, Philadelphia, Pennsylvania; Lewis Katz School of Medicine, Philadelphia, Pennsylvania; Temple University Health System, Philadelphia, Pennsylvania; Temple University Health System, Philadelphia, Pennsylvania; Temple University Hospital, Philadelphia, Pennsylvania

## Abstract

**Background:**

Invasive infection from Group A streptococcus (iGAS) is rising nationally, and we report a significant increase in incidence at an urban, quaternary care health center, which serves the Kensington neighborhood, the epicenter of the opioid crisis in Philadelphia, PA. We examined iGAS infection in the Temple University Health System catchment area

**Methods:**

iGAS was defined as an of streptococcus pyogenes cultured from a previously sterile site. Injection drug use (IDU) is a known risk factor for bacterial infection, including iGAS infection. All blood, sterile fluid, and/or tissue cultures that yielded *S. pyogenes* were identified using the laboratory information system at Temple University Hospital – Main Campus. Two cohorts were compared: January 1, 2021, to December 31, 2021, and January 1, 2019, to December 31, 2019. Electronic health records were reviewed and data pertaining to age, gender, and injection drug use were abstracted. Descriptive statistics were used to summarize findings.

**Results:**

155 cases of iGAS were identified in 2021 (105 of which involved bacteremia) compared to 69 in 2019 (42 of which involved bacteremia), representing a 224% increase overall. Of the cases in 2021, 130 (84%) were Persons Who Inject Drugs (PWID) compared to only 39 (57%) in 2019. PWID with iGAS were younger (median age 35 vs 54 in 2019, 39 vs 53 in 2021) and more likely to be male (57% vs 43% in 2019, 68% vs 32% in 2021). Male patients also had a higher incidence of PWID than female patients (56% vs 44% in 2019 and 64% vs 36% in 2021).

**Conclusion:**

During this same time period, the COVID-19 pandemic added to the ongoing opioid crisis in Philadelphia. The city of Philadelphia publicly reports opioid data, which shows that hospitalizations related to non-fatal opioid overdose have exponentially risen in the past two decades. This also coincides with an increase in the presence of xylazine, an adulterant in the Philadelphia fentanyl supply. Xylazine has been implicated in worsening wounds. Our data supports a concerning association between iGAS and PWID.

**Disclosures:**

**All Authors**: No reported disclosures.

